# Connecting the dots: Kerala’s use of digital technology during the COVID-19 response

**DOI:** 10.1136/bmjgh-2021-005355

**Published:** 2021-07-21

**Authors:** Osama Ummer, Kerry Scott, Diwakar Mohan, Arpita Chakraborty, Amnesty Elizabeth LeFevre

**Affiliations:** 1 Oxford Policy Management, New Delhi, Delhi, India; 2 BBC Media Action, New Delhi, India; 3 Department of International Health, Johns Hopkins Bloomberg School of Public Health, Baltimore, Maryland, USA; 4 International Health, Johns Hopkins Bloomberg School of Public Health, Baltimore, MD, USA; 5 International Health, Johns Hopkins Bloomberg School of Public Health, Baltimore, Maryland, USA; 6 School of Public Health and Family Medicine, University of Cape Town, Cape Town, South Africa

**Keywords:** COVID-19, public health

## Abstract

Digital tools are increasingly being applied to support the response to the ongoing COVID-19 pandemic in India and elsewhere globally. This article draws from global frameworks to explore the use of digital tools in the state of Kerala across the domains of communication, surveillance, clinical management, non-clinical support, and core health system readiness and response. Kerala is considered India’s first digital state, with the highest percentage of households with computers (24%) and the internet (51%) in India, 95% mobile phone penetration, 62% smartphone penetration and 75% digital literacy. Kerala has long been a model for the early adoption of digital technology for education and health. As part of the pandemic response, technology has been used across private and public sectors, including law enforcement, health, information technology and education. Efforts have sought to ensure timely access to health information, facilitate access to entitlements, monitor those under quarantine and track contacts, and provide healthcare services though telemedicine. Kerala’s COVID-19 pandemic response showcases the diverse potential of digital technology, the importance of building on a strong health system foundation, the value of collaboration, and the ongoing challenges of data privacy and equity in digital access.

**Summary box**

- The COVID-19 pandemic’s unprecedented global spread and impact has accelerated interest in digital innovation.
- Kerala’s experience showcases the diverse and innovative ways that digital tools can build on a strong underlying health system to support pandemic response across the domains of communication, surveillance, clinical management, non-clinical support and core health system readiness.
- Digital tools in Kerala were able to proliferate rapidly and help meet diverse citizen needs due to high levels of collaboration and intersectoral response that brought together different levels of government and multiple state departments, engaged the private sector, and harnessed the energy of civil society organisations and community volunteers.
- Digital technology has great potential to strengthen public health measures during pandemics, including to rapidly link citizens to food and mental health support.
- Adequate oversight and community participation remains essential to safeguard citizen privacy and ensure equity.

## Introduction

Kerala, a state in southern India, has been applauded for its proactive response to the ongoing COVID-19 pandemic. Kerala reported its first COVID-19 case on 30 January 2020^1^ and as of early November 2020, has had nearly a half million confirmed cases, comprising 6% of India’s total reported cases.^2^ Despite its high number of cases, Kerala’s case fatality rate (0.4%) is one of the lowest in India (1.5%)^2^ and elsewhere globally, including the USA (2.4%) and China (5.2%).^3 4^ Kerala’s success in attaining low case fatality rates is underpinned by its strong multisectoral response which has been characterised by: widespread testing; treatment and containment, including quarantine centres and designated COVID-19 hospitals; and social protection, including distribution of food and essential supplies (table 1 and box 1). While the continued surge of COVID-19 cases across India has not spared Kerala, as of November 2020, the state has managed to prevent the escalation in cases seen elsewhere, along with widespread food insecurity,^5^ and as a result, continues to be considered a global leader in pandemic response.^6 7^

**Table 1:** COVID-19 cases and outcomes in Kerala as of 8 November 2020

Indicators	Kerala	India (overall)
Population^56^	35 307 000	1 348 616 000
Total confirmed cases^3^	486 109	8 507 754
Active rate^3^	17%	6%
Recovery rate^3^	83%	93%
Case fatality rate^3^	0.4%	1.5%
Tests per million^3^	145 151	88 331
Confirmed cases per million^3^	13 839	6383

Box 1 **Features of Kerala’s COVID-19 pandemic management (as of November 2020)**

**Testing**

- At 145 151 tests per million population, Kerala’s testing per capita is higher than the all-India average (88 331). When compared with countries that have similar gross domestic product per capita ($2900), Kerala’s test per million population is on par with Morocco (121 376), exceeding Vietnam (14 640) and Egypt (9679), and below Bhutan (388 000).^57 58^
- When compared with countries that have similar population, Kerala is on par with Peru (139 188 per million), South Africa (82 556 per million) and Bangladesh (14 634 per million).^59^

**Treatment**

- Kerala established 3585 COVID-19 care centres for quarantine and instituted 503 facilities (304 government, 199 private) with 51 152 beds and 1485 intensive care units for COVID-19 services as of 30 October 2020.^60^
- The state created dedicated COVID-19 sections at different levels of government health facilities and rapidly built or repurposed existing facilities to create 29 dedicated COVID-19 hospitals.^60^

**Surveillance**

- On 30 January 2020, the first case was confirmed in Kerala. The state declared a health emergency on the same day. Following the confirmation of two more cases on 2 and 3 February, the government implemented mandatory symptoms-based screening of all incoming passengers from China.^5^ In response to an initial surge in cases by the first week of March, a mandatory screening for all incoming passengers through road, rail and sea was implemented.^51 61 62^
- The government of Kerala developed a robust systematic contact tracing system.^63^

**Containment**

- The government of Kerala provided clean facilities with adequate social distancing and healthy food in free facilities^2 51^; this approach is in contrast to unsanitary and unsafe quarantine facilities for those who could not pay in some parts of India.^64 65^

**Social protection**

- Under the announced economic support package worth $270 million, more than 5 million vulnerable people received welfare payments of $116 (8500 Indian rupees). Moreover, free dry rations were distributed to all public distribution system card holders.^47^
- Adolescent girls, pregnant women, lactating mothers, and children were provided with food through schools and the existing network of nutrition centres, called Anganwadi centres. Additionally, every day about 400 000 cooked meals were distributed to the poor through existing and newly established community kitchens.^47 51^
- The state instituted about 18 000 camps for the 2.5 million migrant labourers working in Kerala, where they could stay and were provided with food and essential supplies.^47 66^

**Figure 1:**
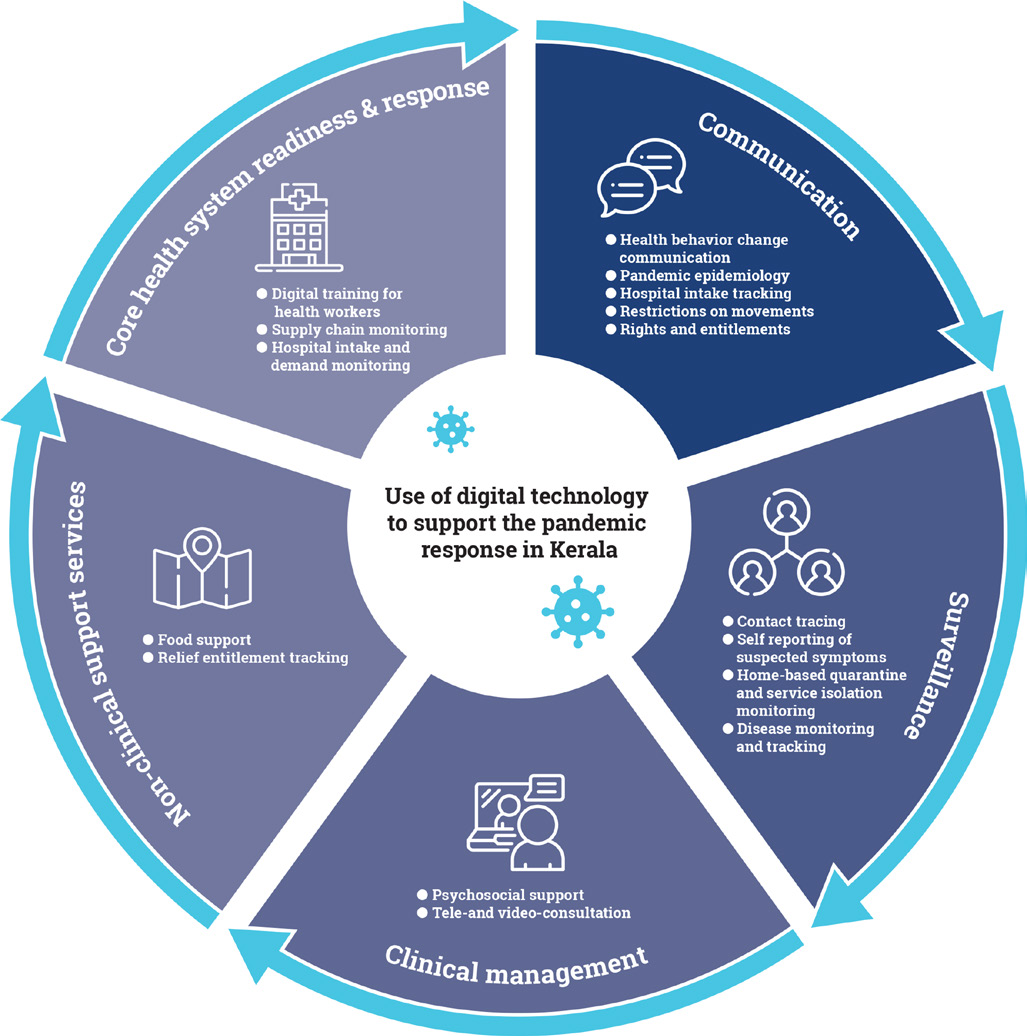
Framework on digital tools for the pandemic response in Kerala.

As India’s first digital state, Kerala has the highest percentage of households with computers (24%) and the internet (51%) in India, 95% mobile phone penetration, 62% smartphone penetration, and 75%digital literacy.^8^ Technology use has been a cornerstone of Kerala’s response to the COVID-19 pandemic. This article presents a mapping of the digital technologies and mobile applications used during Kerala’s COVID-19 response. To identify the applications, in the month of October 2020, the first author searched the Android Play Store for all digital applications available in Kerala related to the COVID-19 response. Among those applications, we selected those that were developed or implemented by the government or in collaboration with the government. We draw from three frameworks on digital tools for pandemic response^9–11^ to group Kerala’s digital tools across the following domains: communication, surveillance, clinical management, non-clinical support, and core health system readiness and response (figure 1). This mapping showcases the range of technologies and tools used (table 2), as well as the intersectoral collaboration involved in their development and the scale and status of each tool’s implementation (table 3). In so doing, this article provides insight into how the use of digital tools can be optimised in the current pandemic response and sustained to enhance routine health services delivery in Kerala, elsewhere in India and globally.

**Table 2:** Overview of digital health solutions, listed in alphabetical order, used in the Kerala COVID-19 pandemic response

Solution name	Description	Digital technology	Digital solution				
			Communication	Surveillance	Clinical management	Non-clinical support	Core health system
1. Arogya Mitra	A chatbot providing information on the Kerala Health Department’s COVID-19 response activities	Web portal	**X**		**O**	**O**	
2. Aarogya Setu	Informing users regarding risks, best practices and relevant COVID-19 advisories	Mobile app	**X**	**X**		**X**	
3. Arogyakeralam	Kerala’s National Health Mission portal, which includes COVID-19-related information	Web portal	**X**		**O**	**O**	**X**
4. BeSafe Tracking	Tracks an individual by device/phone Global Positioning System (GPS)	Mobile app		**X**			
5. BlueTeleMed	Telecounselling and telemedicine	Mobile app			**X**		
6. Break the Chain campaign	Cartoon characters convey important COVID-19 containment messages	Online campaign	**X**				
7. Break the Chain diary	Presents a route map of places visited	Mobile app		**X**			
8. Chiri (smile) telecall	Telecounselling for children’s mental health involving frontline health workers (ASHAs, Anganwadi workers)	Helpline/tele- call	**X**		**X**		
9. CoronaSafe Network—quiz	A multilingual quiz on COVID-19 myths and protective measures	Web portal	**X**				
10. CoronaSafe Network	An open-source disaster management platform	Web portal	**X**	**X**	**X**	**X**	**X**
11. Covid Safety	Bluetooth and GPS-enabled tracker to determine if a user comes into the proximity of a COVID-19-positive person	Mobile app		**X**			
12. COVID-19 Jagratha	Real-time surveillance, care and support for people affected by/quarantined with COVID-19	Mobile app/web portal	**X**	**X**	**O**	**X**	**X**
13. Department of Health and Family Welfare	Information on state health department activities, data visualisation on COVID-19 status, online training modules and communication materials	Web portal	**X**		**O**	**O**	**X**
14. DISHA-1056	A toll-free 24/7 telehealth helpline providing physical and mental health guidance, counselling and information	Web portal/helpline	**X**	**X**	**X**	**O**	
15. Doctor just a phone call away	Telecounselling and telemedicine for police personnel	Helpline, WhatsApp or video consultation			**X**		
16. Emergency Response Support System 112 service	Provides rapid assistance in response to citizen ‘distress signals’ in the form of voice calls, SMSs, email and web requests	Helpline				**X**	
17. eSanjeevani OPD	National teleconsultation service	Mobile app/ web portal			**X**		
18. GoK Dashboard	Government of Kerala dashboard visualising COVID-19 status including daily reporting, quarantine report, test results and hot spots	Web portal	**X**	**X**	**O**	**X**	**X**
19. GoK-Direct Kerala	Shares announcements, updated guidelines and safety tips for visitors to Kerala	Mobile app	**X**				
20. Kerala Battles COVID	Consolidation of COVID-19 management updates for public accessibility	Web portal	**X**		**O**	**O**	**X**
21. Kerala Health Disease Surveillance	Disease surveillance app using phone location; also provides awareness on COVID-19	Mobile app	**X**	**X**	**O**		
22. Kerala Police Home Quarantine Assistance	Coordinates the delivery of non-clinical services to people	Mobile app	**X**	**X**		**X**	
23. Kerala Sannadha Sena (volunteers)	Enrols and coordinates community volunteers	Mobile app/ web portal	**X**			**X**	**X**
24. Kerala Superhero app	Tracks the near real-time location of volunteer assets like ambulance drivers, delivery crews and medical personnel	Mobile app	**X**			**X**	**X**
25. Koode	Enables people under home quarantine to self-report and collate health details daily	Mobile app	**X**	**X**	**O**	**X**	**X**
26. Koode helpline	Telecounselling by Ayurveda doctors	Helpline			**X**		
27. Local self-government Kerala pandemic dashboard	Provides information on local government COVID-19 activities and services	Web portal	**X**			**X**	
28. Ottakkalla Oppamundu (‘not alone with you’)	Provides psychosocial support to children	Helpline			**X**		
29. People Move	Tracks and delivers non-clinical services to people	Mobile app	**X**	**X**	**X**	**X**	
30. Pol-App	Kerala police information programme	Mobile app				**X**	
31. PRASANTHI	Free service provision or directory	Helpline	**X**		**O**	**X**	
32. Project Eagle Eye	Drone tracking of lockdown violations	Drones		**X**			
33. Shops app	Online shopping	Mobile app				**X**	
34. WhatsApp chatbot	Provision of COVID-19 information and directory to services	Mobile app	**X**				**X**
35. Kerala health online training	Educational videos about COVID-19 on YouTube	Mobile app/ web portal	**X**				**X**

X = Provides this service or information directly

O = Links users onward to where this service or information is located

ASHAs, accredited social health activists; SMSs, short message services.

**Table 3:** Actors involved, scale and status of implementation for digital health solutions used in the Kerala COVID-19 pandemic response

Solution name	Actors involved								Scale and status			
	National government		Kerala state government					Private sector	Released on	Downloads	Functionality	
	Health	National Informatics Center	Health	Police	Information technology	Public relations	Other				Functional	Non-functional
1. Arogya Mitra		✔	✔						May 2020	NA		✔
2. Aarogya Setu	✔	✔						✔	Apr 2020	100+ million	✔	
3. Arogyakeralam			✔		✔				NA	NA	✔	
4. BeSafe Tracking				✔	✔			✔	May 2020	1000+		✔
5. BlueTeleMed			✔	✔				✔	Mar 2020	5000+		✔
6. Break the Chain campaign			✔		✔	✔			Mar 2020	NA	✔	
7. Break the Chain diary			✔		✔			✔	Jul 2020	NA		✔
8. Chiri (smile) telecall			✔		✔				Mar 2020	NA	✔	
9. CoronaSafe Network—quiz			✔		✔		✔	✔	Mar 2020	NA	✔	
10. CoronaSafe Network			✔		✔		✔	✔	Mar 2020	NA	✔	
11. Covid Safety	✔								Apr 2020	10 000+		✔
12. COVID-19 Jagratha		✔	✔		✔				Mar 2020	NA	✔*	
13. Department of Health and Family Welfare			✔		✔				NA	NA	✔	
14. DISHA 1056			✔		✔				Mar 2013	NA	✔	
15. Doctor just a phone call away			✔	✔					NA	NA		✔
16. Emergency Response Support System 112 service		✔		✔	✔				NA	NA	✔	
17. eSanjeevani OPD	✔	✔	✔		✔				Jun 2020	100 000+	✔	
18. GoK Dashboard			✔		✔	✔			Mar 2020	NA	✔	
19. GoK-Direct Kerala			✔		✔	✔		✔	Mar 2020	500 000+	✔	
20. Kerala Battles COVID			✔		✔	✔			2020	NA	✔	
21. Kerala Health Disease Surveillance			✔		✔				Mar 2020	1000+	✔	
22. Kerala Police Home Quarantine Assistance				✔	✔			✔	Apr 2020	1000+		✔
23. Kerala Sannadha Sena				✔	✔		✔		Jan 2020	10 000+	✔	
24. Kerala Superhero app			✔	✔	✔	✔			Feb 2020	500+	✔	
25. Koode (citizen centre portal)			✔		✔				Aug 2020	NA		✔
26. Koode helpline			✔		✔				Apr 2020	NA	✔	
27. Local self-government Kerala pandemic dashboard					✔	✔	✔		NA	NA	✔	
28. Ottakkalla Oppamundu (‘not alone with you’)			✔		✔				Jun 2020	NA	✔	
29. People Move by Kerala police				✔	✔			✔	Apr 2020	10+		✔
30. Pol-App				✔					Jun 2020	100 000+	✔	
31. PRASANTHI for senior citizens by the police				✔					Mar 2020	NA		✔
32. Project Eagle Eye				✔					Mar 2020	NA		✔
33. Shops app—online shopping app				✔	✔			✔	Mar 2020	50 000+	✔	
34. WhatsApp chatbot			✔				✔	✔	Mar 2020	NA	✔	
35. YouTube channel— Kerala health online training			✔		✔	✔			Mar 2020	NA	✔	

NA, not applicable.

## Communication

Communication strategies in Kerala have used technology to disseminate health information on disease, risk-mitigation, containment, and the government response, including lockdowns and entitlement programmes.^5^ As shown in table 2, communication was the most common service provided by digital tools developed or implemented by the government or in collaboration with the government in Kerala’s COVID-19 response, with a total of 24 different types of digital communication solutions identified. These solutions included websites, dashboards, web portals, mobile applications, helplines and chatbots as well as social media platforms (box 2).

Box 2 **Key terminology**

- **Chatbots:** virtual assistants that can be deployed on various platforms, both web and mobile. Chatbots can fulfil a variety of tasks such as handling general queries and form filling. They can be implemented on websites and applications to enable bidirectional information flow.
- **Helpline:** a telephone service providing help with problems.
- **Web browsers:** software applications used to browse the internet which enable users to locate and retrieve data across the web. Examples include Safari, Google Chrome and Internet Explorer.
- **WhatsApp Messenger**: cross-platform instant messaging application that allows smartphone users to exchange text, image, video and audio messages for free.
- **Web portal**: acts as a gateway to the world wide web and provides users with a single access point to information.
- **Website:** collection of web pages.

Table 3 showcases that within the first year of the COVID-19 pandemic, almost all of the initial digital solutions that included a communication function have endured. As an early precautionary measure, the state launched an awareness campaign called Break the Chain through television, print media and social media platforms. The government, along with the CoronaSafe Network (an open-source volunteer network), created online quizzes for citizens to get a better understanding of the disease. Initially, the state was disseminating information about COVID-19 through existing health department web portals. However, as it became clear that COVID-19 was a major crisis, the state government rapidly launched a COVID-19-specific dashboard called the COVID-19 Jagratha portal and a mobile application called GoK-Direct. The mobile application, with over 500 000 downloads and a 4.5/5 rating in Google Play Store as of November 2020, emerged as the primary source of reliable information in terms of daily case updates including new information and guidelines issued by the government. These digital platforms disseminated information about government services, including the availability of essential commodities and clinical services. They also connected people to all government orders and guidelines regarding control measures.

Recently, the state launched one more web portal (Kerala Battles COVID) showing the same infographics from the earlier portal and dashboard in the local language (Malayalam). While these multiple portals (Jagratha, GoK-Direct and Kerala Battles COVID) involved some duplication of effort, they maximise reach across languages as well as across different levels of digital capacity and data use.

The state released animated videos on pregnancy care, care for lactating mothers and elderly care during the lockdown period, to fill face-to-face healthcare service gaps. The GoK also used other multimedia platforms such as community radio, an FM campaign and a daily WhatsApp program called POSHAN Vani (Nutrition Voice) to disseminate COVID-19-related information to citizens.^12^ The Kerala state police department’s Social Media Cell and Media Centre created awareness videos, posts and memes, and disseminated them through social media platforms. One video featured police officers dancing to demonstrate handwashing techniques and received over 3.1 million views and nearly 40 000 shares.^13^ The GoK created media surveillance units that monitored news media for issues related to COVID-19. The media surveillance unit would report problems identified in the media to government authorities and would counter fake news by generating press releases.^14^

Civil society organisations also used social media space for communication about the pandemic. For example, Kudumbashree (the empowered women self-help groups) used their existing network of 2 200 000 neighbourhood group members to form 300 000 WhatsApp groups to disseminate government orders, posters and other communication materials on COVID-19 safety measures during lockdown.^5 15^

## Disease surveillance

The state has used a wide range of digital applications for disease surveillance activities such as contact tracing, self-reporting, monitoring home quarantined individuals and enforcing lockdown measures (table 2). We identified 13 digital health solutions to support disease surveillance, including four that focused solely on surveillance: Break the Chain diary and Covid Safety enabled citizens to determine whether they came into contact with someone who tested positive, Project Eagle Eye employed drones to monitor lockdown adherence and BeSafe Tracking shared location data with surveillance agencies to monitor quarantine.

Over half of the technologies that included disease surveillance launched very early in the pandemic, in March 2020, in response to early needs for disease tracking and lockdown enforcement. However, by late 2020, 7 of the 13 digital solutions ceased to function, and an eighth (COVID-19 Jagratha) had dropped the mobile app component, highlighting the evolving nature of the pandemic and limitations on citizen willingness to engage in surveillance activities. In the first 4–5 months of the pandemic, the state government developed route maps for people who tested positive to facilitate contact tracing.^16^ These maps featured a flow chart listing each place, date and time of the visit by that individual. The government then circulated the patient route maps on social media and requested people to contact them through a 24/7 helpline number. The government enlisted multiple helplines including DISHA-1056, district and state corona cell numbers, and the GoK’s corona ‘war room’ landline and WhatsApp number. By September 2020, press reports suggest that the government had stopped preparing route maps because the daily cases were increasing, and it was impossible to trace all cases as many clusters of cases were formed. However, the helplines remained active for citizens to connect with government services.

Beyond the digital solutions that solely focused on surveillance, many popular tools included a surveillance component. During the initial lockdown, the state launched a self-reporting app called Koode; individuals who returned to Kerala from outside the state could input data about their health for 28 days while they were in quarantine. Koode operated through a web application, interactive voice response system, mobile application and telegram (a social media application) as a medium of reporting and ultimately reduced the burden on health workers to follow up with each person in quarantine.^17^ However, when the lockdown ended and cases increased, the application became redundant and is no longer available. The national level government of India developed a mobile application called Aarogya Setu to strengthen surveillance; in Kerala, this application was mainly used by people engaging in interstate travel when lockdowns were eased.

The state used geographic information system and Global Positioning System data from mobile phones, as well as geofencing, social media monitoring, drone footage and security camera footage to track positive cases and define high-risk containment zones.^18–22^ The government also encouraged citizens to use a self-tracking application called Break the Chain diary to track their travel history,^23^ the geotagging option on the mobile phone app COVID-19 Jagratha to flag potential self-quarantine violations,^24 25^ and four other applications related to COVID-19 (Home Quarantine Assistance, BeSafe Tracking, People Move and Covid Safety) that used mobile phone data to track movement. None of the apps were mandatory and few citizens voluntarily submitted to this level of surveillance.

## Clinical management

Kerala used digital technology to provide clinical services related to COVID-19 as well as a wide spectrum of illness through video consultation, e-prescription, telemedicine and psychosocial support. As shown in table 2, nine digital solutions directly provided clinical management, including diagnostics, clinical status monitoring, medical advice and counselling, and another 10 linked people onward to where support could be availed. Table 3 showcases the intensity of intersectoral collaboration within Kerala’s clinical management digital space, with the Kerala state health and information technology departments collaborating on most of these digital tools and further engagement from actors in the police and private sector.

The state introduced the central government’s telemedicine service called eSanjeevani in June 2020 in response to COVID-19 lockdowns. eSanjeevani provides free online teleconsultation and video consultation service through a web portal or mobile application, including specialist and e-prescription service to patients in their homes.^26^ By the end of September 2020, 232 doctors in Kerala had completed over 33 000 teleconsultations, which were the highest rate of consultations by population across all the states.^27 28^ The Kerala police launched a telemedicine platform called Swaraksha Kasaragode in March 2020 to manage health-related emergency calls and have provided medical assistance to over 25 000 people.^20^

The GoK implemented several mental health and suicide prevention digital services in response to COVID-19 including Chiri (smile), a telecounselling programme run by volunteer student police cadets who direct their peers to professional help and Ottakkalla Oppamundu (You are not alone, we are with you), a psychosocial support programme.^29^ Within Ottakkalla Oppamundu, student counsellors and community health workers (accredited social health activists and Anganwadi workers) identified students in need and connected them and their parents to phone counselling under the supervision of psychiatrists, psychiatric social workers and clinical psychologists.^30^ In addition, the state bolstered counselling available for migrant workers, people under quarantine, students and the general public through the existing DISHA-1056 service, a 24/7 telehealth helpline. Between the beginning of the pandemic and November 2020, the state deployed 1376 mental health personnel who made over 5.1 million phone calls to people under quarantine, people with mental illnesses, children with special needs, migrant workers and older adults living alone during the lockdown.^3^

## Non-clinical support services

Kerala’s COVID-19 response has been particularly adept at addressing the non-clinical support needs of citizens under lockdown, such as the need to continue accessing basic household provisions, particularly food, and the need for extra free housing and nutritional support for those who lost their work due to the COVID-19 containment effort. Table 2 showcases 14 digital solutions that sought to directly meet citizens’ non-clinical needs, such as through online shopping portals and volunteer delivery networks, and an additional six that linked onwards to these types of support services. Although not an immediate non-clinical support service, it is noteworthy that Kerala shifted rapidly to an online mode of education for schoolchildren, with over 6000 online classes attended by over 4.3 million students.^31^

The web-based portal and mobile application COVID-19 Jagratha provided information on community kitchens, home delivery of groceries and travel permits.^25^ Kerala’s government dashboard also provided details about the state’s community kitchens by aggregating and reporting data from local bodies on the number of individuals served.^3^ The government also developed the Kerala Sannadha Sena (social service volunteers) app and web portal. The registration of community volunteers is through the web portal and training through the app. The Kerala state police department developed multiple digital strategies for non-clinical support including the ShopsApp for home delivery of essential commodities; Amrutham, a WhatsApp platform to order groceries from local vendors for home delivery and PRASANTHI, a support service for seniors.^18 20^ Furthermore, they allowed citizens to request home delivery of medicine and other essentials through the emergency service 112 hotline. It is striking that the Kerala police were involved in eight digital services for non-clinical support and only four focused on surveillance (table 3).

## Core health system readiness and response

We identified 12 digital tools that supported Kerala’s core health system readiness and response (table 2). These tools served a range of functions to present available hospital capacity, support health worker training and recruitment, identify emergent hot spots and map the status of ambulances. COVID-19 Jagratha, for instance, offered real-time monitoring of vacant beds and allocated patients according to facility capacity. Training videos about COVID-19 were disseminated on YouTube, through WhatsApp and on mobile apps to orient healthcare workers and volunteers.^32^ As of November 2020, the YouTube channel ‘Kerala online health training’ had about 9850 subscribers; many of its videos were endorsed by celebrities. The state health department appointed 276 doctors in a single day following job interviews through video-conferencing.^33^ The state incorporated digital technology in supply chain management: the digital ledger system tracked essential supplies at the district level in both private and public organisations. This helped the state to monitor and control price and supply availability.^34 35^ The COVID-19 response also leveraged digital technologies used to support core health department functions such as procurement and inventory management, human resources, disease surveillance and hospital management information systems.

## Discussion

The government of Kerala has used digital technology in its COVID-19 pandemic response across the domains of communication, surveillance, clinical management, non-clinical support, and core health system readiness and response. These digital tools have communicated health information including COVID-19 prevention and treatment, disseminated lockdown ordinances, supported monitoring and quarantine, linked citizens to medical and non-medical support (including mental health support), and augmented transparency by sharing up-to-date and granular information about COVID-19’s spread, the availability of services such as hospital beds, and the provision of support such as free and subsidised meals. While future research is needed to assess the public health impact of these tools, Kerala’s experience with digital technology presents strong examples of a wide range of use cases. The state’s experience using digital technology during COVID-19 also highlights the value of strong health system foundations and intersectoral collaboration, as well as issues related to privacy and equity.

## Foundations

Digital health technologies are only as good as the services and governance systems that they support. Kerala’s robust COVID-19 response was built on the foundation of their high-performing health system, which has been a longstanding leader in India in terms of financing, human resources and health outcomes.^36^ The government of Kerala drew from previous experience in using digital technologies for emergency preparedness. Kerala experienced extreme flooding and a Nipah virus outbreak in 2018, and in both cases engaged digital tools as part of their emergency response. The flood and Nipah virus responses have been praised as rapid and well coordinated across government departments, and grounded in community participation in rescue and relief works^37 38^; the Nipah epidemic was halted within a month and after only 19 cases.^39^ Social media platforms played a vital role in disseminating credible information and coordinating all government efforts.^40^ There were apps to connect volunteer relief workers to community members in need and dashboards to track the government’s relief grant spending. The police department also used drones to locate stranded people during the flood.^41^ Key digital components of the Nipah response were a 24/7 Nipah helpline, a call centre, contact tracing by telephones, telemental health support, and daily reports on social media, including Qkopy, Arogya Jagratha, Facebook and WhatsApp.^42^ Kerala’s COVID-19 response built on the successes of many of these innovations from 2018.

## Collaboration

Collaboration within government agencies, such as within departments in the Kerala police, between government agencies, and between the government and the private sector and civil society enabled the rapid and effective use of digital tools. Celebrity endorsement strengthened the reach of health communication materials, data pooling from local, district and state level government enabled complete, timely and accurate reporting of the COVID-19 response across the state, and governmental engagement with volunteers from the private sector produced highly functional and easy-to-navigate digital platforms. For instance, Kerala’s Department of Health and Family Welfare and the Kerala State Disaster Management Authority supported an interdisciplinary group of volunteers to create the CoronaSafe Network, an open-source online platform that emerged as one of the most trusted and popular sources of information. The CoronaSafe Network amalgamated data (such as case counts and testing results) from government bulletins and public dashboards and received direct updates from hospitals to produce data visualisations that consolidated pandemic information, including an interactive map of all hospitals and ambulances.

## Privacy

There have been privacy breaches, as well as accusations of government over-reach in relation to Kerala’s use of digital tools to manage COVID-19. Privacy breaches have included leaks of COVID-19 patient-level data from hospitals (both private and public) and leaks of Google maps link that exposed identifiable information about people who underwent contact tracing.^43–45^ Accusations of government over-reach include the unauthorised sharing of citizens’ COVID-19 information with third party organisations as well as digitally enabled contact tracing, geomapping or other surveillance to enforce home isolation and quarantine. The use of digital tools for this type of surveillance can invade individual privacy. The Kerala police initially used call records from people who tested positive for COVID-19 to facilitate contact tracing. However, this use of technology was stopped following protests and legal arguments that it breached data privacy regulations and citizens’ rights to privacy.^46^ The government of Kerala has made all tracking applications voluntary, with the exception of infection reporting, and has set a limit of 14 days to the retention of call records and other individual data. However, debate continues on the appropriate balance between disease surveillance and individual rights.^47^ Best practice policy on data sharing and access has been slow to be implemented in the state and globally.^48 49^ Manual contact tracing became impractical as the numbers of infections increased in August. Had the state put in place a digital method of tracking similar to the ones instituted by some South East Asian countries like Taiwan and Singapore, contact tracing could have continued.^50^ The extent to which societies will agree to some invasion of privacy for greater public good is a function of their trust in government. In the response to COVID-19, Kerala had demonstrated a fairly high level of trust.^51^

## Equity

As essential information and services increasingly move to the digital sphere, there is a risk that existing socioeconomic inequality is increased. Globally, marginalised social groups are least likely to have access to mobile phones, computers and internet and have the lowest digital literacy. Although digital inequality is less pronounced in Kerala than in the rest of India, exclusion remains a concern. Kerala has relatively high internet penetration at 56%, second only to the National Capital Territory of Delhi at 68%; Kerala also has one of the lowest rural–urban and gender digital divides in the country.^52–55^ Nonetheless, 44% of Kerala’s population has no internet access, around 20% of women in Kerala do not own their own phones, and 3% of rural and 2% of urban households do not have any phone in the household at all. Remote tribal areas are particularly likely to lack reliable cellular network and broadband coverage.^52–55^ These digital gaps were mitigated to an extent by civil society support to vulnerable groups who are the most likely to experience lack of access.

## Conclusion

Digital technology initiatives supported Kerala’s COVID-19 response through communication, surveillance, clinical management, non-clinical support, and core health system readiness and response. This analysis showcases the diverse and innovative ways digital tools can support pandemic response and draws attention to the importance of collaboration. With adequate oversight and community participation to safeguard privacy and ensure equity, digital health has great potential to strengthen public health measures.^9^
